# The validity of Iran’s national university entrance examination (Konkoor) for predicting medical students’ academic performance

**DOI:** 10.1186/1472-6920-12-60

**Published:** 2012-07-28

**Authors:** Yasin Farrokhi-Khajeh-Pasha, Saharnaz Nedjat, Aeen Mohammadi, Elaheh Malakan Rad, Reza Majdzadeh, Farshid Monajemi, Ehsan Jamali, Shahryar Yazdani

**Affiliations:** 1 Medical School, Tehran University of Medical Sciences, Tehran, Iran; 2 School of public health, Knowledge Utilization Research Center, Tehran University of Medical Sciences, Tehran, Iran; 3 Center for Educational Research in Medical Sciences, Tehran University of Medical Sciences, Tehran, Iran; 4 Division of Pediatric Cardiology, Department of Pediatrics, Children’s Medical Center (Pediatric Center of Excellence), Tehran University of Medical Sciences, Tehran, Iran; 5 Ministry of Health and Medical Education, Tehran, Iran; 6 National Organization of Educational Testing, Tehran, Iran; 7 Educational development center, Shahid Beheshti University of Medical Sciences, Tehran, Iran

## Abstract

**Background:**

In Iran, admission to medical school is based solely on the results of the highly competitive, nationwide Konkoor examination. This paper examines the predictive validity of Konkoor scores, alone and in combination with high school grade point averages (hsGPAs), for the academic performance of public medical school students in Iran.

**Methods:**

This study followed the cohort of 2003 matriculants at public medical schools in Iran from entrance through internship. The predictor variables were Konkoor total and subsection scores and hsGPAs. The outcome variables were (1) Comprehensive Basic Sciences Exam (CBSE) scores; (2) Comprehensive Pre-Internship Exam (CPIE) scores; and (3) medical school grade point averages (msGPAs) for the courses taken before internship. Pearson correlation and regression analyses were used to assess the relationships between the selection criteria and academic performance.

**Results:**

There were 2126 matriculants (1374 women and 752 men) in 2003. Among the outcome variables, the CBSE had the strongest association with the Konkoor total score (*r =* 0.473), followed by msGPA (*r =* 0.339) and the CPIE (*r =* 0.326). While adding hsGPAs to the Konkoor total score almost doubled the power to predict msGPAs (*R*^2^ = 0.225), it did not have a substantial effect on CBSE or CPIE prediction.

**Conclusions:**

The Konkoor alone, and even in combination with hsGPA, is a relatively poor predictor of medical students’ academic performance, and its predictive validity declines over the academic years of medical school. Care should be taken to develop comprehensive admissions criteria, covering both cognitive and non-cognitive factors, to identify the best applicants to become "good doctors" in the future. The findings of this study can be helpful for policy makers in the medical education field.

## Background

“Konkoor”, derived from the French word *concours* (meaning competition), is a familiar word for Iranians, as each year hundreds of thousands of high school graduates compete in this extremely difficult exam to enter public universities. The Konkoor was first conducted 46 years ago, in an attempt to introduce a single, common test for the entire pool of national candidates for higher education. Through its relatively long history, the Konkoor has not undergone any significant revisions [1]. While public universities are tuition-free, there is a private chain of universities in Iran named Azad University that charges high fees while having a lower credibility than the public universities [2]. This university administers its own entrance exam, which is very similar to the Konkoor. The Konkoor is a comprehensive, multiple-choice exam with four choices and only one correct answer for each question. The exam covers common high school topics and takes place once a year in 5 different academic streams of “experimental sciences”, “mathematics and physics”, “human sciences”, “fine arts”, and “foreign languages” [3].

As the sole criterion for student admissions into universities, the Konkoor creates psychological and social problems, such as anxiety among the prospective examinees and their parents in Iran, a society in which education is a major determinant of class mobility [1,3]. Furthermore, there are even greater challenges for the educational system. As the Konkoor is so stringent, students are so heavily focused on preparing for the Konkoor in their last years of high school that other aspects of higher-order mental functioning, such as creativity and critical thinking, may be neglected [4,5]. In addition, test preparation has become the focus of teaching in schools, which has turned schools into centers for examination cramming. This is an important factor that prevents a move away from traditional methods toward better educational standards of teaching and assessment [1,3].

Medicine is among the hundreds of fields of study for which high school graduates compete on this extensive exam. In Iran, medical students typically commence their studies right after high school, with neither preliminary higher education nor the adequate mental and personal maturity necessary to choose to study medicine [6]. In addition, because of several social, economical and cultural factors, the study of medicine is among the first priorities of students, and therefore, the best ranked candidates, according to their Konkoor scores, are admitted into the public medical schools [7]. The 7-year medical education program at Iranian universities is divided into an initial 2.5-year basic science or pre-clinical stage, followed by a period of clinical training, which lasts a further 3 years. After those 5.5 years, there are 1.5 years of internship, during which the students practice at ‘university hospitals’ and work under the supervision of residents and fully licensed staff physicians.

Apart from the problems resulting from admitting medical students directly upon graduation from high schools and without any experience in medicine, the nature of the Konkoor is a matter of concern as the sole criterion for medical student admission in the country. Worldwide, predictive validation studies are carried out to develop screening and selection methods to accept the best students into medical schools [8-14]. These studies conclude that prior academic performance account for only part of variance in medical school performance. The predictive validity of the medical school admissions tests in North America and Australia range from small to medium. In these countries, these tests are supplemented by other cognitive and non-cognitive factors [8-14]. However, to the best of our knowledge, there have been no studies on the Konkoor’s validity for predicting the academic success of medical students. Given the concerns that the selection process for medical education programs in Iran may not be optimal, we decided to investigate the extent to which Konkoor scores predict success in medical school. This study reports the association of the Konkoor scores with subsequent student performance in Iranian medical schools.

## Method

### Study sample and data

The subjects consisted of the 2003 cohort of matriculants at all 38 public medical schools in Iran. Following participation in the 2003 Konkoor, the high-ranked applicants were allowed to select their preferred medical schools, based on their ranks on the Konkoor. We followed this cohort from their entrance to medical school through internship. The follow up period was from 2003 to 2009.

### Predictor variables

Preadmission information included in the study was retrieved from the database of the National Organization of Educational Testing (NOET) and consisted of the following.

**High school grade point average (hsGPA).** In this report, hsGPA indicates the average grades of the final 3 years of high school courses. We included hsGPAs in our study because university admission requirements have recently been expanded to include hsGPAs, in addition to the Konkoor [15]. However, hsGPAs were not included in the admission requirements of the cohort of students whose data were used in this study. The maximum attainable score of hsGPA is 20.

### Konkoor scores

High school graduates who want to enroll in public medical schools must take a version of the Konkoor that is made up of 8 subsections: The first 4 are non-science courses (Persian language, Arabic language, religious studies and a foreign language — usually English), and the next 4 are science courses (mathematics, biology, physics and chemistry). The crude scores of each subsection are calculated by multiplying the number of correct responses by 3, then subtracting the number of incorrect responses and last, dividing that product by the total number of questions in the subsection. Because there is difference among the subsections of one examination with regard to the level of difficulty, the obtained crude scores are adjusted across all the subsections. Once adjusted for the respective level of difficulty in the exam, each subsection receives a weighted score, and the Konkoor total score is generated by summing the weighted scores across subsections using the following equation [15]:

(1) $$Konkoor\;total\;score=\frac{\left(4*Per+2*Ara+3*Rel+2*For\right)+3*\left(2*Mat+4*Bio+2*Phy+3*Che\right)}{44}$$

where *Per* stands for Persian language, *Ara* for Arabic language, *Rel* for religious studies, *For* for foreign language, *Mat* for mathematics, *Bio* for biology, *Phy* for physics, and *Che* for chemistry. As is evident from the equation, science courses are given 3 times as much weight as non-science courses. All 8 subsection crude scores (without adjusting), total non-science subsection (sum of 4 non-science crude sub-scores), total science subsection (sum of 4 science crude sub-scores) and the Konkoor total score were used in this study. The crude scores were used so that the results of the article can be comparable to equivalent studies in other countries. Konkoor total score was also used in this study as the medical student selection is based on this variable.

### Outcome Variables

Data on the academic performance of medical students were gathered from the Ministry of Health and Medical Education (MOHME) database and were added to the database containing their preadmission information. This data consisted of the following.

Comprehensive Basic Sciences Exam (CBSE) scores. The CBSE is a centralized, nationwide multiple-choice examination that is held at the end of the fifth semester of medical school and focuses on the understanding and application of basic science areas that are relevant to medical education. Students have to pass this examination to proceed to the clinical stage.

Comprehensive Pre-Internship Exam (CPIE) scores. The CPIE is also a centralized, nationwide multiple-choice examination that is held at the end of the clinical stage and assesses the clinical knowledge of the medical students. CPIE tests only knowledge and does not have a clinical skills component. Passing this exam qualifies a medical student to begin his or her internship.

Medical school grade point average (msGPA). Medical school GPAs were computed from all courses completed prior to internship and were weighted according to the number of course hours. In the clinical stage, the examinations mostly assess both clinical skills and clinical knowledge. The clinical skills are usually assessed in different ways like Standardized Patients.

### Analysis

The Konkoor, CBSE and CPIE are centralized, nationwide examinations, and scores are reported on the same scale for all students. In addition, given the standard curriculum for all of the medical schools in the country [16], we collapsed msGPAs across the schools, and holistic analyses were carried out for the entire body of national students.

To answer the questions related to the predictive validities of the Konkoor and hsGPAs, correlations between the predictor variables and outcome measures were calculated using Pearson’s correlation coefficient. In order to examine the predictive power of different combinations of preadmission variables, multiple linear regression was performed for each outcome variable with 8 different predictor sets:

1 hsGPA alone;

2 Konkoor non-science crude sub-scores (Persian language, Arabic language, religious studies and foreign language);

3 Konkoor science crude sub-scores (mathematics, biology, physics and chemistry);

4 All Konkoor crude sub-scores (Persian language, Arabic language, religious studies, foreign language, mathematics, biology, physics and chemistry);

5 All Konkoor crude sub-scores and hsGPA;

6 Total non-science subsection and total science subsection;

7 Konkoor total score alone; and

8 Konkoor total score and hsGPA.

For each equation, all the variables in each predictor set were entered simultaneously into a stepwise regression procedure where the significance of each variable which is introduced into the model is assessed until the best fitting model is obtained. Associations were considered significant when *P* values < 0.05. All analyses were performed using SPSS statistical software (version 18.0, SPSS Inc., Chicago, Ill., USA).

As with other validation studies, Cohen's calibration was used to guide interpretation of the results in this report [8,11]. In this regard, the effect size of a coefficient from 0.100 to 0.200 was considered to be “small”, from 0.300 to 0.500 was considered to be “medium” and 0.500 or greater was considered to be “large”.

This study received approval from the local ethics committee and was performed in accordance with the Declaration of Helsinki. Individual student consent was not obtained as this was a retrospective analysis of an anonymised database.

## Results

Table 1 shows the descriptive statistics for variables used in this study. For the 2003 academic year, there were 2126 matriculants (1374 women and 752 men). Relying on the NOET database, Konkoor scores were available for all students, whereas hsGPAs of only 1161/2126 (54.61%) students could be located. At the same time, the MOHME database contained CBSE scores for 1759/2126 (82.74%) students, CPIE scores for 1886/2126 (88.71%) students, and msGPAs for 1822/2126 (85.70%) students. Complete data only was available for 800 (37.63%) of participants.

**Table 1:** Descriptive statistics for variables used in the study

	**Mean**	**SD**	**Max**	**Num**
***hsGPA***	18.3675	1.48171	20	1161
***Per***	81.694	10.1369	100	2126
***Ara***	67.206	19.5505	100	2126
***Rel***	81.588	10.2619	100	2126
***For***	77.049	16.6000	100	2126
***NS***	307.53636	43.097171	400	2126
***Mat***	45.033	21.8005	100	2126
***Bio***	79.067	13.9110	100	2126
***Phy***	55.286	20.0649	100	2126
***Che***	75.733	14.0190	100	2126
***S***	255.11863	56.059116	400	2126
***Tot***	9869.07	851.538	---	2126
***CBSE***	137.45	20.753	200	1759
***CPIE***	127.02	18.281	203	1886
***msGPA***	15.2401	1.49175	20	1822

SD = standard deviation; Max = maximum; Num = number of participants for whom data was available; hsGPA = High school grade point average (see the text for more details); Per = Persian language; Ara = Arabic language; Rel = Religious studies; For = Foreign language; NS = Total non-science subsection (see the text for more details); Mat = Mathematics; Bio = Biology; Phy = Physics; Che = Chemistry; S = Total science subsection (see the text for more details); Tot = Konkoor total score (see the text for more details); CBSE = Comprehensive Basic Sciences Exam; CPIE = Comprehensive Pre-Internship Exam; msGPA = Medical school grade point average (see the text for more details).

Correlations between the predictor and outcome variables are shown in Table 2. All the correlations presented in Table 2 are statistically significant. The Konkoor predictive validity coefficients for the CBSE were the greatest among the outcome variables, and other than mathematics and physics, the Konkoor correlation coefficients for msGPAs were greater than those for the CPIE. Konkoor total score had a medium predictive validity coefficient effect size for the CBSE (*r =* 0.473), the CPIE (*r =* 0.326) and msGPA(*r* =0.339). Other than religious studies (*r* = 0.284) and foreign language (*r* = 0.289), other Konkoor sub-score correlations associated with the CBSE would be classified as medium in size. (range: *r =* 0.301 to 0.402) Conversely, other than the correlation coefficients of Arabic language (*r* = 0.323) and biology (*r* = 0.300) with msGPAs, all of the Konkoor sub-score predictive validity coefficients were small (range: *r =* 0.138 to 0.261) for both the CPIE and msGPAs. The hsGPA predictive validity coefficient is greatest for msGPAs (*r =* 0.445), followed by the CBSE (*r =* 0.331) and CPIE (*r =* 0.225), respectively. Comparing the correlation coefficients of science and non-science courses with outcome variables, showed that CPIE had a better correlation with science courses, and msGPA had a better correlation with non-science courses. The correlations of science and non-science courses with CBSE are close to each other in magnitude.

**Table 2:** Correlations between predictor variables and academic performance

	**hsGPA**	**Per**	**Ara**	**Rel**	**For**	**NS**	**Mat**	**Bio**	**Phy**	**Che**	**S**	**Tot**
***CBSE***	.331^**^	.314^**^	.376^**^	.284^**^	.289^**^	.425^**^	.301^**^	.402^**^	.319^**^	.336^**^	.414^**^	.473^**^
***CPIE***	.225^**^	.200^**^	.221^**^	.138^**^	.187^**^	.257^**^	.230^**^	.255^**^	.245^**^	.233^**^	.303^**^	.326^**^
***msGPA***	.445^**^	.261^**^	.323^**^	.247^**^	.222^**^	.355^**^	.177^**^	.300^**^	.202^**^	.241^**^	.277^**^	.339^**^

** *P* < α = 0.001.

hsGPA = High school grade point average (see the text for more details); Per = Persian language; Ara = Arabic language; Rel = Religious studies; For = Foreign language; NS = Total non-science subsection (see the text for more details); Mat = Mathematics; Bio = Biology; Phy = Physics; Che = Chemistry; S = Total science subsection (see the text for more details); Tot = Konkoor total score (see the text for more details); CBSE = Comprehensive Basic Sciences Exam; CPIE = Comprehensive Pre-Internship Exam; msGPA = Medical school grade point average (see the text for more details).

Table 3 presents the results of multiple regression analyses using 7 combinations of preadmission variables, which were mentioned in the methods section, to predict 3 outcome variables: A1-8 for the CBSE, B1-8 for the CPIE and C1-8 for msGPAs. For each model standardized coefficient, the relative independent contribution of the predictor variables and variance-explained statistics (*R*^*2*^) are presented.

**Table 3:** Regression statistics for different models of the relationship between predictor variables and academic performance (A1-8 for the CBSE, B1-8 for the CPIE and C1-8 for msGPA)

**Model**	**R**^**2**^	**hsGPA**	**Per**	**Ara**	**Rel**	**For**	**NS**	**Mat**	**Bio**	**Phy**	**Che**	**S**	**Tot**
*CBSE*													
A1	.109	.331^**^											
A2	.185		.122^**^	.229^**^	.139^**^	.078^**^							
A3	.189							.147^**^	.284^**^	NI	.097^**^		
A4	.233		.082^**^	.162^**^	.103^**^	NI		.094^**^	.228^**^	NI	NI		
A5	.229	.135^**^	.094^**^	.141^**^	.125^**^	NI		.107^**^	.129^**^	NI	NI		
A6	.213						.271^**^					.240^**^	
A7	.223												.473^**^
A8	.213	.159^**^											.367^**^
*CPIE*													
B1	.050	.225^**^											
B2	.064		.110^**^	.132^**^	NI	.079^**^							
B3	.093							.112^**^	.132^**^	.077^**^	.069^*^		
B4	.098		.066^**^	.068^*^	NI	NI		.095^**^	.127^**^	.074^*^	NI		
B5	.116	.107^**^	.078^*^	NI	.063^*^	NI		.107^**^	.093^**^	.093^*^	NI		
B6	.99						.115^**^					.233^**^	
B7	.106												.326^**^
B8	.126	.094^**^											.307^**^
*msGPA*													
C1	.197	.445^**^											
C2	.133		.102^**^	.230^**^	.137^**^	NI							
C3	.097							.053^*^	.231^**^	NI	.083^**^		
C4	.153		.075^**^	.190^**^	.102^**^	NI		NI	.161^**^	NI	NI		
C5	.246	.342^**^	.088^**^	.144^**^	.093^**^	NI		NI	NI	NI	NI		
C6	.129						.301^**^					.084^**^	
C7	.114												.339^**^
C8	.225	.352^**^											.192^**^

** *P* < α = 0.01; * *P* < α = 0.05; NI = Not included in the final model. (As they did not contribute significantly to model’s fit).

hsGPA = High school grade point average (see the text for more details); Per = Persian language; Ara = Arabic language; Rel = Religious studies; For = Foreign language; NS = Total non-science subsection (see the text for more details); Mat = Mathematics; Bio = Biology; Phy = Physics; Che = Chemistry; S = Total science subsection (see the text for more details); Tot = Konkoor total score (see the text for more details); CBSE = Comprehensive Basic Sciences Exam; CPIE = Comprehensive Pre-Internship Exam; msGPA = Medical school grade point average (see the text for more details).

For the outcome variables, explanations of variance by Konkoor scores were greatest for the CBSE, followed by msGPAs and the CPIE, respectively. All Konkoor sub-scores combined (A4) and Konkoor total score alone (A7) explained 23.3% and 22.3% of the variation in students’ performance on the CBSE, respectively. When hsGPAs along with Konkoor scores (A5, A8) were applied for predicting the CBSE, *R*^*2*^ values declined slightly, compared with when Konkoor scores were used alone (A4, A7). The predictive power of Konkoor non-science sub-scores (*R*^*2*^*=* 0.185) was similar to that of science sub-scores (*R*^*2*^*=* 0.189) for performance in the CBSE. In predicting CBSE, validity coefficients obtained for total science and non-science subsections were close to each other (A6).

The CPIE was the least predictable of the outcome variables. Different combinations of preadmission variables could explain, at most, 12.6% of the variance in CPIE scores. Adding hsGPAs to regression models that already included Konkoor scores (B5, B8) predicted little better than Konkoor scores alone (B4, B7). Although both had weak predictive validity, Konkoor science sub-scores appeared to have slightly stronger correlations with CPIE scores (*R*^*2*^*=* 0.093) than did non-science sub-scores (*R*^*2*^*=* 0.064). Validity coefficients obtained for total science subsection was more than two-fold higher than that of non-science (B6).

In contrast to the CPIE, Konkoor non-science sub-scores were better predictors of msGPAs (*R*^*2*^*=* 0.133) than science sub-scores (*R*^*2*^*=* 0.097). Validity coefficients obtained for total non-science subsection was more than three-fold higher than that of science (C6). The results, also, indicated that when all Konkoor sub-scores were allowed into stepwise regression analyses for the prediction of msGPA (C4), among science sub-scores, only scores in biology remained in the model, and none of the Konkoor science sub-scores remained when hsGPAs were added to the equation (C5). Compared with other outcome variables, hsGPAs were relatively strong predictors of msGPAs. Table 3 shows that the percentage of variance explained in msGPAs nearly doubled when hsGPAs were added to Konkoor scores as predictors (C5, C8), compared with when Konkoor scores were used alone (C4, C7).

## Discussion

We used 3 sets of scores as outcome variables in this article. We used the CBSE as an indicator of academic performance for the first 5 semesters of basic science or the pre-clinical stage. CPIE scores were used to explore the relationship between selection criteria and performance near the end of the medical program. Furthermore, medical students take examinations throughout their educational program, and we used the weighted average grades for all courses taken before internship (msGPA) as an indicator of average performance throughout the medical education program.

The results obtained showed relatively weak correlations between the predictors and academic performance. Konkoor total score and sub-scores, as the sole admission criteria, were relatively poor predictors of medical students’ academic performance, especially for CPIE scores. The findings of this study indicate that the Konkoor is not a consistent predictor, and its predictive validity declines over the academic years in medical school. In addition, because there is considerable variation in the predictive validity of different science and non-science subtests, revisions should be considered in the weighting system or in limiting the use of some subtests. Of course, there were discrepancies among outcome variables regarding their predictability by Konkoor subsections. While religious studies and foreign languages had the least predictive validity of the Konkoor subsections for the CBSE and CPIE, mathematics and physics showed the weakest relationship among predictor variables with msGPA. Conversely, biology maintained a strong relationship with all 3 outcome variables. Evaluating the association between Konkoor scores and other outcome variables can pave the way for better interpretation of the predictive validity of the Konkoor’s subsections in the future. Although not included in the admission criteria for the cohort of students whose data were used in this study, hsGPAs increased predictive values for the CPIE, as did msGPAs, in particular, when added to regression models that already included Konkoor scores. However, this combination slightly reduced the explanatory power of the model for predicting CBSE scores. The discrepancy in the predictive validity of hsGPA between outcome variables could be attributed to different methods of examination. Both hsGPA and msGPA are average course grades of students over a relatively long period of time. Both of these variables employ similar methods (multiple tests over a long period), whereas the Konkoor, CBSE and CPIE are centralized exams that are mostly taken only once by each student in his or her life. Perhaps different skills are needed to excel in these 2 different methods of evaluation. A similar rationale has been proposed to explain the difference in the predictive validity of the MCAT for medical school and licensing exam performance [8].

Selecting a limited number of students from a large pool of applicants, to produce “good doctors”, is the ultimate goal of the medical schools’ admission committees. This is of particular importance in an educational system in which less than 1% of the total number of applicants across the country are finally accepted into medical schools [7]. Studies that examine the capacity of the selection criteria to assure future successful performance are necessary to help admissions committees make sound and evidence-based decisions. There have been few studies investigating the relationship between Konkoor scores and medical students’ performance in Iran, all of which have been published in local journals. In one study, successful medical students (those without a history of dropping out and with a medical school GPA greater than 15 out of 20) performed better on the Konkoor science subsections compared with their unsuccessful counterparts (with a history of dropping out) [17]. In another study, there was no significant relationship between the rank on the Konkoor and the total score of critical thinking among 89 students at Isfahan University of Medical Sciences [18]. The role of critical thinking has been highlighted in medical education, and it has been recommended to consider training in critical thinking as a part or pre-requisite of the medical curriculum [19].

Various studies have investigated the validity of the North American Medical College Admission Test (MCAT) and the Graduate Australian Medical School Admissions Test (GAMSAT) for predicting medical school performance, and these studies have assessed the extent to which these exams supplement the power of other medical school admissions criteria. The results of previous studies on the predictive validity of the MCAT have shown that correlations between this exam and academic results vary mostly from 0.3 to 0.6 [8-10,20]. Moreover, a review of literature on the value of the GAMSAT in predicting medical school performance indicated that this exam alone is relatively poor at predicting academic performance [12,13]. Nevertheless, it should be considered that these college entrance tests are used in conjunction with other admissions requirements, such as undergraduate GPA and interviews, which supplement the predictive power of these exams. As a result, these selection tools, in combination, are good predictors of students’ subsequent performance [10,14] Compared to the MCAT and especially the GAMSAT, the Konkoor by itself is not a poor predictor of medical school performance. However, it is not supplemented by any other criteria, and this fact renders all admissions criteria relatively poor predictors of performance at Iranian medical schools. A difference between Konkoor and these admission tests is that, as mentioned in the method section, negative marks are awarded for Konkoor which can mask the true candidate ability.

Among the MCAT subtest scores, the biological sciences has the largest predictive validity for measures of medical school performance [8]. In predicting performance on the medical board licensing examination measures, the biological sciences and verbal reasoning subtests of MCAT have better predictive validity than other subsets [8]. In British studies, also, it has been documented that A level chemistry and biology are good predictors of performance in basic medical science examinations [21]. Among the sections of GAMSAT, section III (on reasoning in biological and physical sciences) is most strongly associated with year 1 academic performance [14]. In the present study, the science sub-scores – especially biology – showed the largest predictive validity for all outcome variables. Although chemistry showed large correlation coefficients in correlation analyses, in most regression models it was not included in the final model.

Besides cognitive abilities, medical schools generally agree that non-cognitive abilities are important contributors to the ability of students to become competent physicians, and hence, it is not sufficient to admit students solely on the basis of academic achievement. In recent years, there have been many attempts, such as the Multiple Mini-Interview, to develop assessment tools that are capable of predicting the non-cognitive qualities of the candidates [22,23]. Although evidence has emphasized the importance of non-cognitive characteristics to the admissions process, these characteristics have played no role in the admission of medical students in Iran.

Recently, there have been some reforms in an attempt to improve medical education in Iran, such as graduate entry to medical schools [6,7,16] Furthermore, in an attempt to improve the admissions procedure, hsGPAs have in the past 3 years been added to the Konkoor in the admissions process for all fields of study, including medicine [15] Although these reforms are valuable, they need to be systematically assessed in the future.

An important limitation of the present study was that the validity coefficients were not corrected for range restriction and criterion unreliability, and hence, the observed relationship may be an underestimate of true validity. As mentioned above, applicants to all fields of study in the stream of “experimental sciences” (including medicine) participate in one examination on one day (392073 applicants for the 2003 Konkoor on experimental sciences). After reporting the results, eligible students are allowed to declare their top 100 field-department-university priorities in the order of their preferences. They can select any field of study in the stream of the Konkoor in which they have participated. Thus, the definition of the *true* applicant pool for medicine is not clear with this exam, and therefore, correction for restriction is not possible. At the same time, as described in previous studies [24], assuming that all the applicants in the stream of “experimental sciences” are the applicant pool for this study would likely result in an overestimation in that the total testing sample across all fields of study in this stream is generally more variable (has greater standard deviations) than *true* applicants to medicine. In addition, the data from MOHME show that the CBSE and CPIE have acceptable reliabilities, which hover around 0.95 in recent years. As a result, we assumed that criterion unreliability may not be the case in this study, and we did not perform the respective corrections. Missing data, especially for students’ high school GPAs, was another limitation of this study. Most of the missing data are due to the shortcomings of MOHME database. This is “missing at random” and thus causes no bias. Notably, with regard to Konkoor scores, there was no significant difference between the group of applicants with hsGPA data and the group of applicants for whom data on hsGPA were missing (*P* value = 0.130). Of course, a minor part of the missing data is due to delays and attrition which can confound the results. Unfortunately, we have no precise information on this error. The outcome measurements in our study were knowledge-based examinations that test the students' recall of information. Consequently, we were not able to assess the clinical skills of medical students and their relationship with admissions criteria as important measurements of medical students’ performance.

This nationwide study followed one cohort of students from entrance to medical school through internship. We used different outcome variables to provide information about the relationship between Konkoor scores and academic performance in different learning phases. In contrast to many predictive validation studies [9,14,20], the predictor and outcome variables used in this study were not school-dependent, and therefore, we were able to conduct a single analysis for the entire national population. Nevertheless, the predictive validity of the Konkoor needs to be evaluated with other outcome variables. Work should be done on the validity of admissions criteria for predicting residency national exams and other domains, such as practical skills, clinical performance, professionalism and job satisfaction. These measurements are of particular importance because sound criterion measurements for non-cognitive domains is an issue that most studies have not addressed [25].

## Conclusion

Admission criteria for medical schools should provide acceptable predictive validity of performance in medical school during both the preclinical and clinical years. This recommendation gains even more importance in medical schools with undergraduate programs, as applicants are selected for a longer process of education, compared with graduate programs. The Konkoor alone, and even in combination with hsGPA, has limited predictive value that could be augmented by the introduction of other assessments. We believe that there is a need to develop admission requirements with improved validity that can supplement the Konkoor as a criterion for admission to medical schools. These admission requirements should include both cognitive and non-cognitive factors to identify well-qualified students for the curriculum.

## Competing interests

This study is based on the MD-MPH dissertation of the first author. The authors declare that they have no competing interests.

## Authors' contributions

YF 1) designed the study, acquired the data and analyzed and interpreted the data; 2) drafted the article. SN was the research supervisor of the study and 1) designed the study, assisted in data analysis and interpretation of the data; 2) revised the article critically for important intellectual content. AM 1) made substantial contributions to the acquisition of data; 2) revised the article critically for important intellectual content. EMR, RM, FM, EJ and SY made substantial contributions to the acquisition of data; 2) revised the article critically for important intellectual content. All the authors approved the final manuscript.

## Pre-publication history

The pre-publication history for this paper can be accessed here:

http://www.biomedcentral.com/1472-6920/12/60/prepub
